# Development of a gene-editing strategy to overcome genetic intractability in *Lactobacillus johnsonii*

**DOI:** 10.1128/jb.00100-26

**Published:** 2026-06-29

**Authors:** Keerthikka Ravi, Nicole R. Falkowski, Gary B. Huffnagle

**Affiliations:** 1 Department of Molecular, Cellular & Developmental Biology, University of Michigan 1259, Ann Arbor, Michigan, USA; 2 Division of Pulmonary and Critical Care Medicine, Department of Internal Medicine, University of Michigan 1259, Ann Arbor, Michigan, USA; 3 Mary H. Weiser Food Allergy Center, University of Michigan 1259, Ann Arbor, Michigan, USA; 4 Department of Microbiology & Immunology, University of Michigan, Ann Arbor, Michigan, USA; The Ohio State University, Columbus, Ohio, USA

**Keywords:** transformation efficiency, markerless, *upp*, 5-fluorouracil, beta-galactosidase

## Abstract

Genetic editing in lactobacilli is often constrained by their robust defense mechanisms against foreign DNA and low transformation efficiency, resulting in pronounced species- and strain-specific variability in the performance of genetic tools. In this study, we developed a markerless gene editing system for *Lactobacillus johnsonii* MR1, a Jackson mouse isolate with a closed genome and well-documented host immunomodulatory properties. Initial testing revealed that existing transformation and gene-editing protocols produced low transformation efficiency, poor reproducibility, and exhibited poor plasmid stability in *L. johnsonii* MR1. To address these challenges, we established a counterselectable marker-based approach utilizing the *upp* gene, encoding uracil phosphoribosyltransferase, to enable efficient selection of recombinant colonies following plasmid curing. We generated an in-frame isogenic *upp* mutant strain (*L. johnsonii* KR09) and constructed a temperature-sensitive counterselectable plasmid, pG+DualMarker3, derived from pG+Host9 and carrying the *Lactobacillus acidophilus upp* cassette. Deletion of *upp* conferred resistance to 5-fluorouracil (5-FU), which was reversed upon plasmid introduction. We demonstrated the efficacy of this system by creating an in-frame deletion of *lacLM*, encoding beta-galactosidase, and restoring the wild-type phenotype through complementation. Additionally, 5-FU resistance enabled differentiation and tracking of inoculated versus indigenous *L. johnsonii* populations *in vivo* in mouse models. This robust gene-replacement strategy enhances genetic accessibility in *L. johnsonii* MR1 and establishes a foundation for mechanistic studies of host colonization and host-microbe interactions in mouse models.

**IMPORTANCE:**

Understanding the complex interplay between beneficial gut microbes and their hosts demands tools that enable precise gene editing of the microbes. *Lactobacillus johnsonii* MR1, a rodent isolate with host health beneficial properties, has posed major obstacles to gene editing due to its high strain heterogeneity and limited compatibility with existing molecular tools. In this study, we establish a robust markerless gene replacement system employing a counterselectable marker in *L. johnsonii* MR1, enabling efficient and targeted genome modifications. By expanding the genetic toolbox available for *L. johnsonii*, our approach provides a solid foundation for future investigations into microbe-microbe and host-microbe interactions and facilitates the engineering of strains with customized health benefits.

## INTRODUCTION

Gene editing in lactobacilli remains challenging, largely due to their robust defense mechanism against foreign DNA (1). This limits the availability of genetic tools for these species. Furthermore, genetic editing in lactobacilli is often species and strain-dependent (2). *Lactobacillus johnsonii* is a commensal bacterium prevalent in the gastrointestinal tract of humans, rodents, birds, and pigs (3). The host health beneficial properties of *L. johnsonii* strains, including pathogen exclusion, enhancement of epithelial barrier function, and modulation of host immune responses, have positioned this bacterium as an attractive candidate for therapeutic and dietary interventions (3–9). The strain heterogeneity in *L. johnsonii* suggests that transformation and gene-editing approaches must be tailored to the specific isolate (10). To advance our understanding of the underlying mechanisms behind these several health beneficial properties, the ability to genetically manipulate this species becomes indispensable.

Traditionally, genome editing in lactobacilli has relied on homologous recombination systems, such as the pORI system (11–13). The pORI system is a two-plasmid system with a helper plasmid and an integration plasmid. The helper plasmid encodes an antibiotic marker and a temperature-sensitive *repB,* while the integration plasmid encodes a different antibiotic marker, lacks *repA,* and encodes the homologous insert for gene editing. The temperature-sensitive RepA protein is functional only in a narrow range of temperatures, that is, at nonpermissive temperatures, often above 37°C, RepA is inactivated, and the plasmid can no longer replicate. By growing the cells with specific antibiotics and temperature, the integration plasmid can be diluted out, and a stable mutant genotype can be acquired. While this system has been successfully adapted in many lactobacilli species (14, 15), the need to sequentially transform multiple plasmids, followed by temperature-dependent curing of the plasmids, limits the efficiency and speed of gene editing.

The use of counterselectable markers in bacteria has emerged as a powerful tool to overcome these hurdles and streamline mutant identification following recombination events (14, 16, 17). Typically, the counterselectable marker is on the vector, and hence, the presence of the vector makes the cells susceptible to selective pressure. By killing cells that retain the marker after the second recombination event, stable mutants can be efficiently selected without extensive screening. Additionally, because marker-carrying cells are selectively killed, the same marker can be reused in subsequent rounds of genetic manipulation, enabling multiple gene deletions or insertions within a single strain.

In this study, we report on the development of a gene editing system in *L. johnsonii* MR1. This strain was isolated from Jackson mice, has a complete closed genome on NCBI, and has been successfully used in several experiments reporting the immunomodulatory ability of this strain (6, 18–21). We developed an optimized protocol for high-efficiency transformation and established a counterselection system based on *upp* (uracil phosphoribosyl transferase) for efficient markerless gene replacement. Using these tools, we generated targeted gene deletions in *L. johnsonii* and complemented mutant strains, demonstrating both the practical utility and reliability of our approach. We further validated that *upp* deletion does not compromise *in vivo* colonization fitness, providing a foundation for advanced genetic studies in host-microbe interactions and probiotic development.

## RESULTS

### Optimizing *L. johnsonii* transformation efficiency

The first step to setting up a gene editing system is improving transformation efficiency (22). Low transformation efficiency limits the number of cells that can take up foreign DNA, making molecular cloning and genome editing labor-intensive and time-consuming. By increasing transformation efficiency, the likelihood of recovering recombinant clones increases, thereby accelerating the construction of mutant libraries.

We began by assessing the reproducibility of existing protocols (23, 24) in *L. johnsonii* MR1. Protocols I and II (Horn et al. [23] and Palomino et al. [24]) vary in species tested, the preparation of competent cells, and the electroporation parameters. While the transformation efficiency of the (23) protocol is not reported, it has been successfully used in *L. johnsonii* FI9785 for gene deletion studies, using pG+Host9 plasmid. The Palomino et al. (24) protocol has been reported to yield a transformation efficiency of 6.0 × 10^6^ CFU/µg DNA when transforming pG+Host9 into *Lactococcus lactis*. However, in *L. johnsonii* rodent isolate MR1, the transformation efficiency for pG+Host9 plasmid using protocol I was 8.8 × 10^1^ CFU/µg DNA and protocol II was 1.9 × 10^2^ CFU/µg DNA. In addition to low efficiency, both approaches showed poor reproducibility in *L. johnsonii* MR1. The fall in transformation efficiency when moving to a new system (different strain or species) observed here highlights the fundamental hurdle faced when setting up a gene editing toolbox for lactobacilli species. The low transformation efficiency suggested that adapting the existing protocols for transformation in *L. johnsonii* was not feasible.

To develop a new transformation protocol, using a stepwise optimization approach, we tested all the parameters that are known to impact transformation efficiency. These include (i) growth media and conditions, (ii) electroporation buffer, and (iii) electroporation parameters. We started by optimizing the growth conditions of the cells. We tested the effect of supplementing the growth media with varying concentrations of glycine, ranging from 0.5% to 2%. We observed that supplementing the media with glycine increased the transformation efficiency, with the best results observed at 2% glycine concentration (Fig. S1A). Sucrose magnesium chloride electroporation buffer (SMEB) is commonly used for the lactobacilli species (25–27). For *L. johnsonii,* we observed better transformation efficiency for 3× SMEB, with a 10 times increase in efficiency (data not shown). Finally, we tested different electroporation parameter settings for resistance and voltage. We tested four resistance settings—200 Ω, 400 Ω, 800 Ω, and INF (data not shown)—as well as four voltage settings—7.5, 9, 10, and 12.5 kV/cm (Fig. S1B). Consistent and optimal transformation efficiency was observed when electroporation was done at 10 kV/cm, 400 Ω, and 25 µF, yielding a transformation efficiency of 3.9 × 10^3^ CFU/µg DNA.

**Fig 1:**
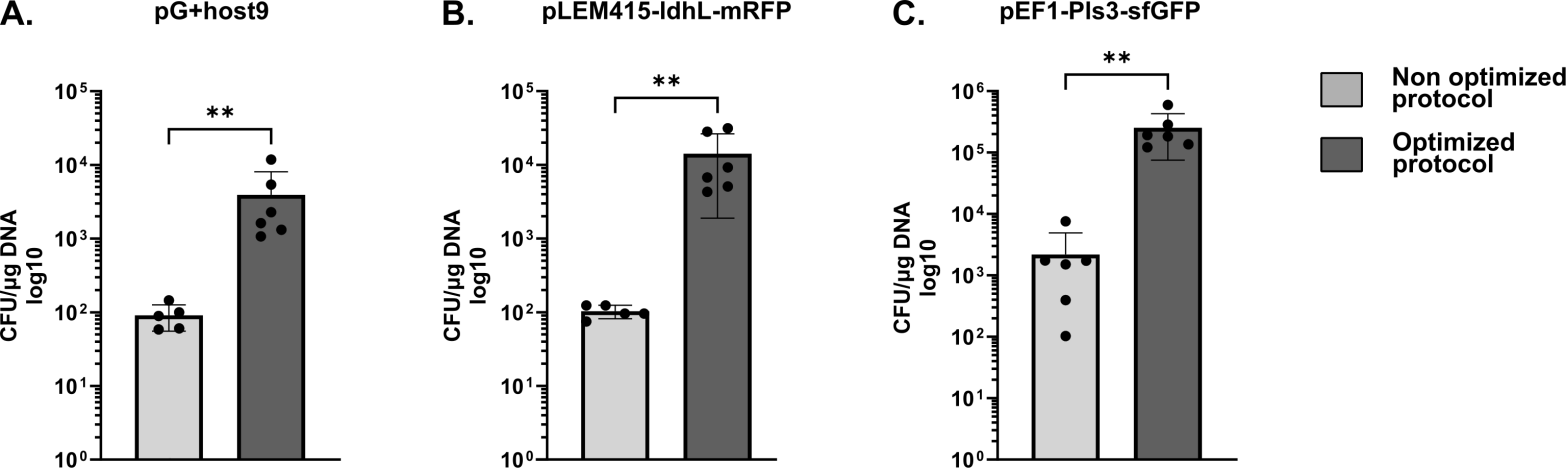
Optimized transformations of various plasmids in *L. johnsonii*. Three plasmids, pG+Host9 (**A**), pLEM415-ldhL-mRFP (**B**), and pEF1-Pls3-sfGFP (**C**), were transformed into electrocompetent *L. johnsonii* MR1 cells that were prepared either using the non-optimized protocol or the optimized protocol. The optimized electroporation procedure is efficient across all three plasmids. Data were collected from three independent experiments performed in duplicates. The Mann-Whitney test was run to assess a significant difference in transformation efficiencies between the two protocols (*P < 0.05, **P <0.005).

Next, we tested the efficiency of the optimized protocol when transforming three plasmids, pG+Host9, pLEM415-ldhL-mRFP1 (28), and pEF1-Pls3-sfGFP (29) in *L. johnsonii* MR1 (Fig. 1). pG+Host9 and pLEM415-ldhL-mRFP1 are low-copy plasmids, while pEF1-Pls3-sfGFP is a high-copy plasmid. Our optimized protocol gave a 44×, 135×, and 115× increase in efficiency for pG+Host9, pLEM415-ldhL-mRFP1, and pEF1-Pls3-sfGFP, respectively. Notably, there was a significant difference in transformation efficiency between plasmid types. These data suggest that our optimized protocol can be used to successfully transform any plasmid into *L. johnsonii* rodent isolate.

### Selection and testing of uracil phosphoribosyl transferase (*upp*) as a counterselectable marker in *L. johnsonii*

**Fig 2:**
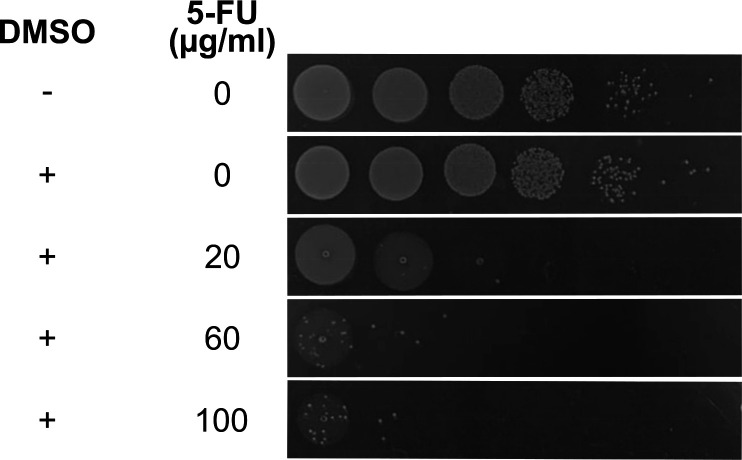
Determining minimum inhibitory concentration of 5-FU for *L. johnsonii*. Exponential-phase culture of *L. johnsonii* MR1 was serially diluted and plated on MRS media supplemented with either DMSO or different concentrations of 5-FU dissolved in DMSO. After 24 h incubation, 100 µg/mL concentration of 5-FU yielded the least number of colonies on the plate (representative, reproducible image from at least three experiments testing inhibitory concentrations of 5-FU for *L. johnsonii*).

To increase the efficiency of a markerless gene replacement approach, we decided to use a counterselectable marker, selecting *upp* as the counterselectable marker candidate. Uracil phosphoribosyl transferase is involved in the pyrimidine salvage pathway, where it catalyzes the conversion of uracil and 5-phospho-alpha-D-ribose 1-diphosphate (PRPP) into uridine 5′-monophosphate (UMP) and diphosphate. Presence of *upp* makes the cells susceptible to a toxic uracil analog, 5-fluorouracil (5-FU), whereas the absence of the gene confers resistance to 5-FU. To determine the sensitivity of *L. johnsonii* MR1 to 5-FU, fresh culture at an OD600 of 0.5 was serially diluted and plated on MRS media supplemented with different concentrations of 5-FU, ranging from 0 to 100 mg/mL. Plates were incubated at 37°C for 24 h under anaerobic conditions. Cells were additionally also plated in equal amounts of solvent, dimethyl sulfoxide (DMSO), to verify that the observed toxicity is due to 5-FU. Least growth was observed at a concentration of 100 µg/mL, where on plating roughly 10^5^ cells, only 15 colonies appeared (Fig. 2). The presence of DMSO did not affect the growth of cells, suggesting that the presence of 5-FU in the media inhibited the growth of *L. johnsonii*. Thus, a final concentration of 100 µg/mL of 5-FU was used for all counterselection plating.

### Development of a Δ*upp L. johnsonii* strain

**Fig 3:**
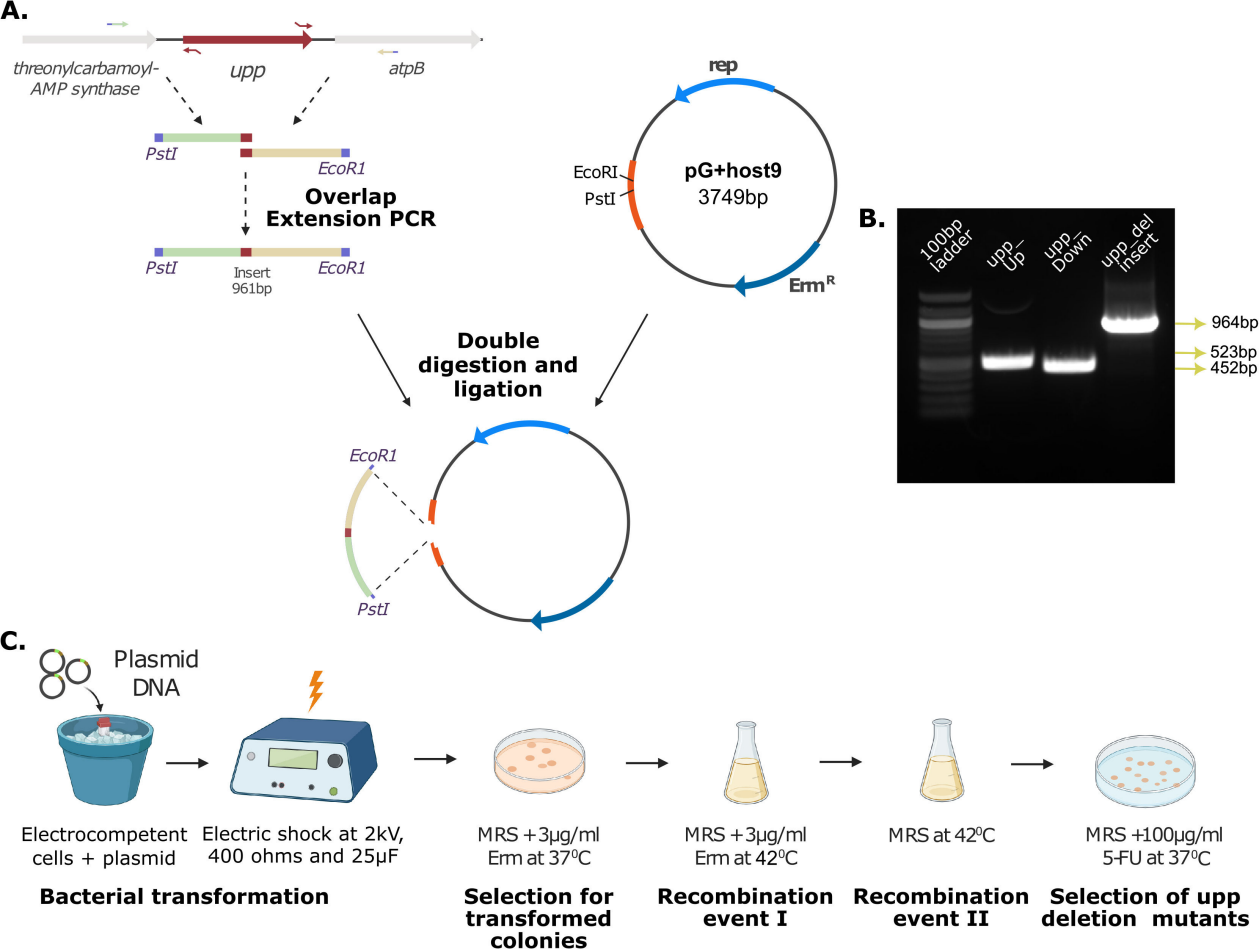
Construction of an *L. johnsonii* MR1 Δupp strain. (**A**) Schematic representation of creating an insert for *upp* deletion using overlap extension PCR and cloning into pG+Host9 plasmid. (**B**) Gel image showing the PCR amplified *upp* flanking fragments (upp_up, upp_down) and the final fused insert after overlap extension PCR (upp*_*del insert). (**C**) Schematic representation of transformation and mutant selection procedure in *L. johnsonii*. Schematics created with Biorender.com.

To develop a *L. johnsonii* strain compatible with counterselectable marker-based gene replacement, the first step was to create a *upp* isogenic mutant of *L. johnsonii*. For this, the temperature-sensitive plasmid pG+Host9 was used and the plasmid constructs for homologous recombination-based gene deletion were cloned as demonstrated in Fig. 3A. The upstream and downstream sequences of the *upp* gene were amplified through PCR and combined to form the insert through overlap extension PCR (Fig. 3B). The insert was then cloned into pG+Host9 through double digestion and ligation. *E. coli* Top10 was used as the intermediate cloning host. Once the deletion plasmid sequence was confirmed, the plasmid was transformed into electrocompetent *L. johnsonii* MR1. Successful *upp* gene deletion mutants in *L. johnsonii* MR1 were selected by plating transformed colonies on 5-FU plates following recombination events (Fig. 3C).

**Fig 4:**
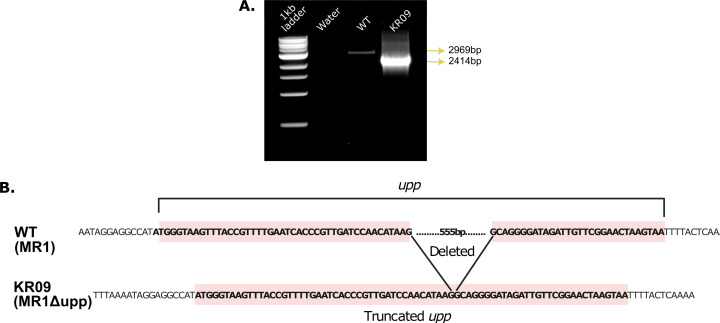
Genotypic and phenotypic characterization of *L. johnsonii* KR09 (MR1 Δupp). (**A**) Gel image showing the PCR amplicons set up using primers targeting the upstream and downstream region of *upp* in wild-type strain (MR1) and *upp* deletion strain (KR09). The results indicate a 500 bp difference in amplicon size. (**B**) Whole genome sequencing-based confirmation of in-frame deletion of *upp* gene. *L. johnsonii* KR09 has a clean 555 bp in-frame deletion. Compared to the wild-type strain, KR09 does not have any other large insertion and deletion segments.

**Fig 5:**
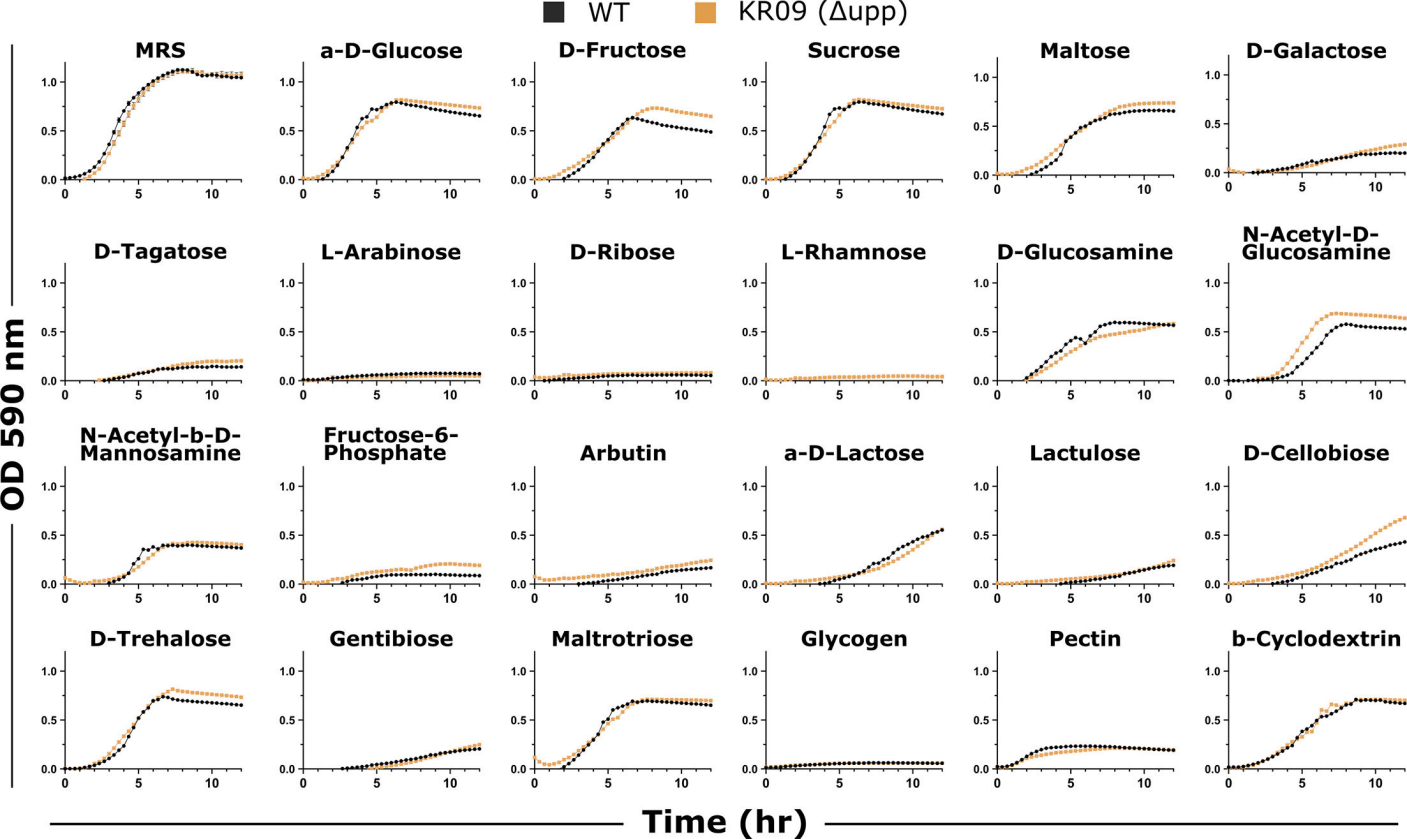
Phenotypic characterization of *L. johnsonii* KR09 (MR1 Δupp). Comparison of carbohydrate utilization profile of *L. johnsonii* KR09 and the wild-type strain. Using BIOLOG, the utilization of 190 carbohydrates was tested (Figs. S2 and S3). The figure shown here is representative, drawn from multiple experiments.

Next, sequencing and phenotyping approaches were taken to confirm a clean in-frame deletion of *upp* in the 5-FU-resistant mutants. Initial confirmation of gene deletion was done through colony PCR, using primers targeting 1 kb upstream and downstream sequence. Of the 5-FU-resistant colonies screened by PCR, one isolate (KR09) was selected for subsequent in-depth analysis and experimentation. There is an approximate 500 bp difference in the PCR product size between the wild-type strain (MR1) and Δ*upp* strain (KR09) (Fig. 4A). Additionally, through whole genome sequencing, we confirmed a clean in-frame deletion of *upp* in *L. johnsonii* KR09 compared to *L. johnsonii* MR1 (Fig. 4B). Furthermore, no additional insertion or deletion sites were identified in the Δ*upp* strain (KR09). There was no significant difference in growth rate between the wild-type and mutant strain when grown anaerobically or aerobically at 37°C in MRS media (Fig. 5). Finally, to ensure that the deletion of *upp* did not affect carbon metabolism in KR09, we tested and compared the ability of both *L. johnsonii* MR1 and KR09 to utilize 190 different carbon sources (Fig. 5, Fig. S2 and S3). Of the 190 carbon substrates tested, we did not see a loss or gain in utilization of carbon substrate for growth under the conditions tested.

### Development of a *upp* expression plasmid for *L. johnsonii*

**Fig 6:**
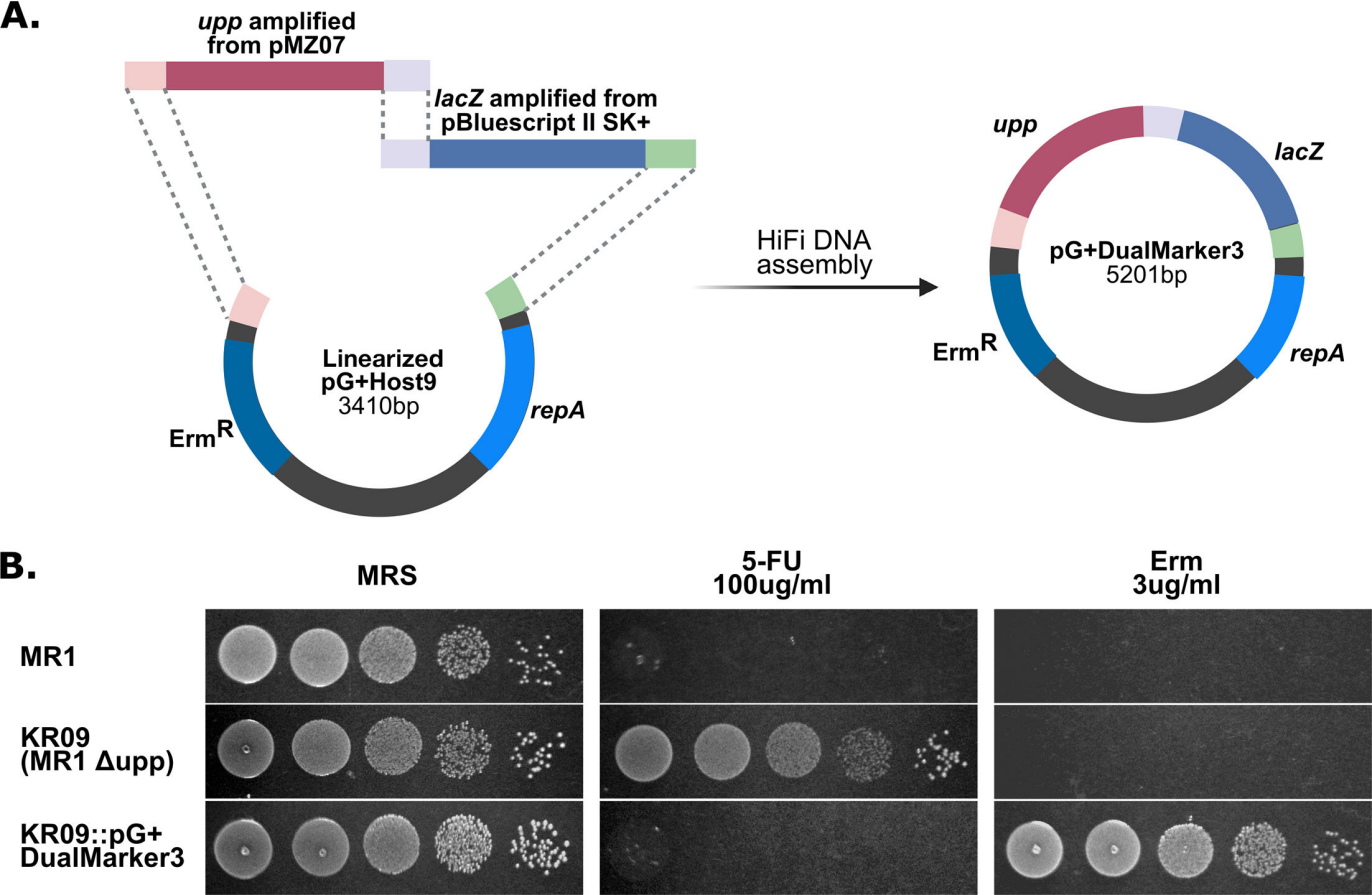
Construction of the pG+DualMarker3 plasmid. (**A**) Schematic representation of the construction of pG+DualMarker3 plasmid. *L. acidophilus upp* expression cassette acquired from pMZ7 plasmid and *lacZ* from pBluescriptSK were cloned into pG+Host9 vector using Gibson Assembly. Created with Biorender.com. (**B**) Successful *upp* complementation using serial dilution spotting on selective plates. *L. johnsonii* MR1 (wild type), *L. johnsonii* KR09 (MR1 Δupp), and *L. johnsonii* KR09 transformed with pG+DualMarker3 were serially diluted and plated on MRS media or MRS media supplemented with 5-FU. or erythromycin (Erm). *L. johnsonii* MR1 was susceptible to both 5-FU and Erm. Deletion of *upp* conferred resistance to 5-FU, while the presence of pG+DualMarker3 in *L. johnsonii* KR09 rescued the wild-type phenotype.

The next step in developing the counterselectable marker-based gene replacement system is to construct a vector that expresses the counterselectable marker. The expression of the counterselectable marker, in this case, *upp,* in Δ*upp* would make the cells susceptible to 5-FU killing, while cells lacking the vector will be resistant. We used pG+Host9 as the temperature-sensitive plasmid for creating this construct. pTRK669, another common temperature-sensitive plasmid often used in lactobacilli, was found to be unstable in *L. johnsonii* (data not shown). To construct the *upp* expression plasmid, we first PCR-amplified the pG+Host9 backbone sequence, including the antibiotic resistance gene. The presence of temperature-sensitive *repA* in pG+Host9 makes it a suitable candidate for gene replacement experiments, as shown above. The *Lactobacillus acidophilus upp* expression cassette was amplified from the pMZ7 plasmid (26), and the *lacZ* fragment containing multiple cloning sites was amplified from the pBluescriptII SK(+) plasmid to enable blue/white screening in future cloning experiments. Both the *upp* cassette and *lacZ* fragment were amplified with 20–30 bp overlaps homologous to adjacent fragments, as indicated in Fig. 6A. The three fragments—plasmid backbone, upp cassette, and lacZ—were assembled using Gibson Assembly, and the resulting plasmid, pG+DualMarker3, was cloned into *E. coli*. Following sequence confirmation of the plasmid, we assessed pG+DualMarker3 *upp* expression and its compatibility with *L. johnsonii* KR09. To do this, we compared the growth of *L. johnsonii* MR1, KR09, and KR09 transformed with pG+DualMarker3 (KR09::pG+DualMarker3) on various selective media (Fig. 6B). All three strains grew on non-selective MRS media. *L. johnsonii* MR1, which carries the wild-type *upp* gene, was susceptible to 5-FU. *L. johnsonii* KR09, lacking the *upp* gene, was resistant to 5-FU. Introducing the *upp*-expressing pG+DualMarker3 plasmid into *L. johnsonii* KR09 restored 5-FU sensitivity, confirming plasmid-driven *upp* expression. This confirms that the strain and plasmid developed are compatible for counterselectable marker-based gene replacement.

### Utilizing counterselection to delete of *lacLM* in *L. johnsonii* MR1

**Fig 7:**
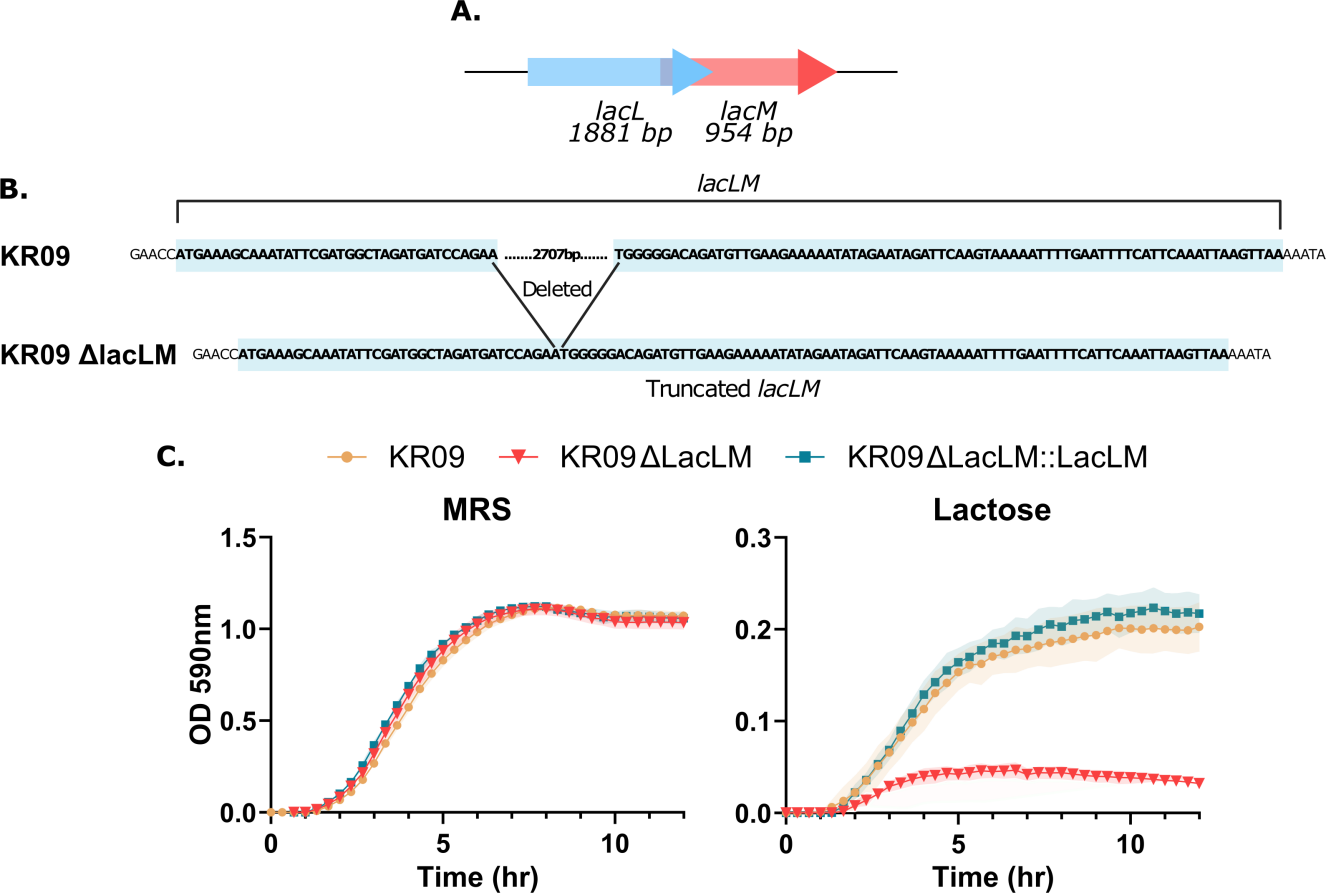
Deletion of the heterodimeric beta-galactosidase genes (*lacLM*) in *L. johnsonii*. (**A**) Schematic representation of unidirectional heterodimeric beta-galactosidase genes in *L. johnsonii, lacLM*. Created with Biorender.com. (**B**) Sequence confirmation of a clean in-frame deletion of *lacLM* in KR09 ΔlacLM. (**C**) Phenotype confirmation of *lacLM* deletion. *L. johnsonii* KR09, KR09 ΔlacLM*,* and KR09 ΔlacLM complemented with *lacLM* were grown in regular MRS media or sugar-free MRS media supplemented with 10% lactose as the sole carbon source (*n* = 4). Deletion and insertion of *lacLM* had no impact on the growth of the three strains in regular MRS media. However, when lactose is the sole carbon source, only *L. johnsonii* KR09 and KR09 ΔlacLM::lacLM strain were able to grow. KR09 ΔlacLM was unable to grow using lactose.

To test the feasibility of the *upp*-based counterselection gene replacement system in *L. johnsonii* KR09, we created an isogenic mutant of the heterodimeric beta-galactosidase *lacLM,* in *L. johnsonii* KR09. Similar to *Lactiplantibacillus plantarum*, *L. johnsonii* has two forms of beta-galactosidase, a heterodimeric form encoded by *lacLM* (Fig. 7A) and a homodimer form encoded by *lacZ* (30). In *L. plantarum, lacLM,* and not *lacZ*, was shown to be essential for growth using lactose as the sole carbon source. By deleting the *lacLM* genes in *L. johnsonii* KR09, our objective is to see whether lactose utilization ability is impeded.

The plasmid deletion construct for homologous recombination-based gene deletion was cloned similar to the *upp* deletion construct. The upstream and downstream fragment sequence of *lacLM* was combined through overlap extension PCR and cloned into the multiple cloning site in pG+DualMarker3 plasmid through restriction digestion and ligation. Successful transformants were identified through blue-white screening followed by PCR and plasmid sequencing confirmation. The plasmid construct was then transformed into electrocompetent *L. johnsonii* KR09. Transformed cells were grown at different selective pressures to trigger recombination events and were finally plated on MRS plates supplemented with 5-FU to select for colonies that no longer carry the plasmid. Confirmation of *lacLM* deletion in *L. johnsonii* KR09 was done using colony PCR primers, targeting the upstream and downstream sequences of *lacLM*. We observe an approximate 2.7 kb drop in band size in KR09 ΔlacLM compared to the wild-type strain (data not shown). A clean in-frame deletion of *lacLM* was confirmed in the mutant strain by whole genome sequencing (Fig. 7B).

To investigate the phenotypic effect of the loss of *lacLM* genes, *L. johnsonii* KR09 and KR09 ΔlacLM were grown in regular MRS media and sugar-free MRS media supplemented with 10% lactose. *L. johnsonii* KR09 was able to grow in both media. However, KR09 ΔlacLM was only able to grow in MRS and not sugar-free MRS media supplemented with lactose (Fig. 7C). To further validate that the loss of lactose utilization is due to the loss of *lacLM* genes, the genes were reintroduced in the KR09 ΔlacLM strain. The insertion of the *lacLM* genes was done similarly to the gene deletion described previously, using pG+DualMarker3 plasmid. Complementation of *lacLM* genes, in the Δ*lacLM* strain, rescued the growth of cells in the lactose media (Fig. 7C). Overall, these experiments demonstrate (i) that *lacLM* plays an essential role in lactose utilization in *L. johnsonii*, similar to *L. plantarum,* and (ii) the *upp* counterselection-based gene replacement approach developed here for *L. johnsonii* was able to effectively delete and insert sequences into the chromosome.

### Analysis of gastrointestinal fitness in Δ*upp L. johnsonii* KR09

**Fig 8:**
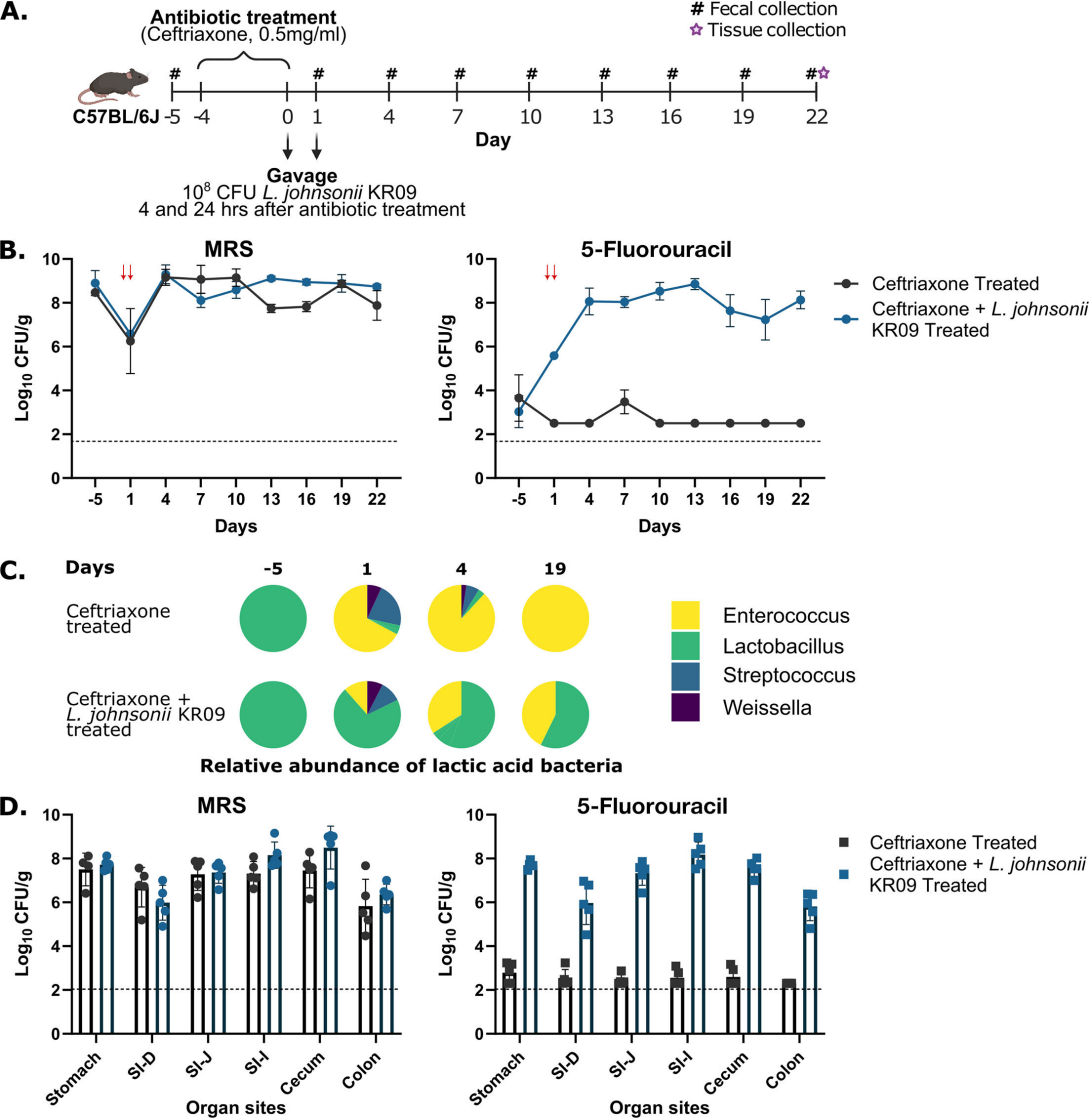
Analysis of gastrointestinal fitness in Δ*upp L. johnsonii* KR09. (**A**) Schematic representation of the *in vivo* colonization experiments. C57BL/6J mice (*n* = 5 per group), following antibiotic treatment were either left to recover (ceftriaxone treated) or given two oral gavage doses of *L. johnsonii* KR09 (ceftriaxone + *L. johnsonii* KR09 treated). The gut microbiome composition of these mice was monitored for 21 days using plating and 16S microbiome analysis. (**B**) Levels of lactic acid bacteria in fecal samples collected periodically during the course of the animal experiment. Samples were plated in regular MRS media and media supplemented with 5-fluorouracil. The orange arrows represent the time points of *L. johnsonii* KR09 oral gavage. (**C**) Relative abundance of lactic acid bacteria in fecal samples in ceftriaxone-only and ceftriaxone + *L. johnsonii* KR09-treated cohort. (**D**) Levels of lactic acid bacteria in different niches of the GI tract, monitored by plating samples in MRS media and media supplemented with 5-FU.

Next, we investigated whether the deletion of the *upp* gene affected the ability of *L. johnsonii* KR09 to colonize the GI tract of mice. *L. johnsonii* KR09 is a derivative of the mouse isolate *L. johnsonii* MR1; hence, we sought to confirm that the deletion of *upp* did not affect the ability of the strain to colonize the GI tract of mice. Furthermore, we hypothesized that the resistance to 5-FU in *L. johnsonii* KR09 also provided a unique opportunity to track the levels of the bacterium in a mixed microbial population by using 5-FU selective plating, since other bacteria in the microbiome should be sensitive to 5-FU. The *in vivo* experiment was set up as shown in Fig. 8A. C57BL/6J mice were administered the broad-spectrum cephalosporin antibiotic ceftriaxone for 4 days to deplete the endogenous gut microbiome as previously described (31). After the cessation of antibiotic treatment, one group was left to recover naturally, while the other group received two oral gavage doses of *L. johnsonii* KR09. Fecal samples were collected periodically over the subsequent 20 days to monitor gut microbiome composition and lactic acid bacteria (LAB) abundance. At the end of the study, GI tract tissue samples were harvested. All samples were plated on either standard MRS media or MRS supplemented with 5-FU.

To investigate the ability of *L. johnsonii* KR09 to persist in the GI tract of antibiotic-treated mice, we analyzed the LAB community composition in fecal samples. Prior to ceftriaxone treatment, all mice exhibited high baseline LAB levels (Fig. 8B). Plating and colony PCR analyses established *L. johnsonii* as the dominant *Lactobacillus* species. Following antibiotic administration, both groups experienced a pronounced reduction in LAB populations. In the ceftriaxone-only group, Enterococcus became the dominant LAB following a single day of recovery (Fig. 8C). In contrast, the ceftriaxone + *L. johnsonii* KR09 group showed detectable levels of both Enterococcus and Lactobacillus species. Selective plating on 5-FU confirmed that the Lactobacillus species colonizing the second group was specifically *L. johnsonii* KR09. Notably, by the end of the study, the gut microbiome composition of both groups did not return to baseline, as observed through 16S rRNA microbiome analysis. Throughout the course of the experiment, Enterococcus remained the dominant LAB in the ceftriaxone-only group, whereas the ceftriaxone + *L. johnsonii* KR09 group maintained consistent levels of both Enterococcus and *L. johnsonii*.

Next, to assess the ability of *L. johnsonii* KR09 to colonize the various niches of the GI tract, we analyzed the LAB community composition in the stomach, small intestine, colon, and cecum. Total LAB levels in these niches were comparable between both treatment groups when assessed on non-selective MRS plates (Fig. 8D). However, when samples were plated on 5-FU selective media, LAB counts in the ceftriaxone-only group fell below the limit of detection. In contrast, the ceftriaxone + *L. johnsonii* KR09 group exhibited colony-forming unit (CFU) counts on 5-FU-selective plates that mirrored those seen on non-selective media for all GI niches examined. These data indicate that *L. johnsonii* KR09 can stably colonize the murine GI tract following antibiotic-mediated depletion of the native lactobacilli population.

Collectively, these results demonstrate that *L. johnsonii* KR09, which lacks *upp*, is able to colonize the gastrointestinal tract of mice at levels comparable to the parent strain *L. johnsonii* MR1. This supports the utility of this strain for *in vivo* experimentation, enabling reestablishment of *L. johnsonii* in the gut post-antibiotic treatment. Moreover, the 5-FU resistance of *L. johnsonii* KR09 provides a valuable tool for *in vivo* studies, facilitating the analyses of both host-microbe and microbe-microbe interactions within complex microbial communities.

## DISCUSSION

The most significant finding of the work presented here is the development and validation of the *upp*-based, temperature-sensitive plasmid system, pG+DualMarker3, in the *upp* mutant background strain. This system enables efficient selection of transformed cells using the erythromycin resistance marker (*erm*) and counterselection via 5-FU sensitivity conferred by the *upp* gene. The effectiveness of this system is further strengthened by our systematic optimization of the transformation protocol, thereby significantly improving genetic accessibility.

The concept of using *upp* as a counterselectable marker has been employed in various LAB. Like in *L. johnsonii* MR1, work done in *Lactobacillus casei* and *L. lactis* showed that combining *upp* counterselection with a temperature-sensitive plasmid allowed for marker-free gene deletions without affecting growth or stability (17). In *L. acidophilus, Lactobacillus gasseri,* and *Enterococcus faecalis*, *upp* counterselection marker coupled with ORI plasmid system has been successfully used for precise gene editing (14, 15, 32).

Nevertheless, the use of *upp* as a counterselectable marker is not without challenges. If the host strain has a redundant uracil salvage pathway, 5-FU may retain residual toxicity even in *upp* mutants (33). Early studies in *L. lactis* demonstrated that *upp* deletion alone conferred only limited 5-FU resistance and that targeted mutations in thymidine phosphorylase or thymidine kinase were necessary for robust counterselection (34). *L. johnsonii* lacks thymidine phosphorylase and so would be unable to convert uracil to dUMP through deoxyuridine, suggesting that *upp* is solely responsible for sensitivity to 5-FU. In line with this, our *L. johnsonii* KR09 strain exhibited robust resistance to 5-FU upon *upp* deletion. Furthermore, *upp* is conserved among strains of *L. johnsonii*, suggesting a favorable metabolic context across all strains for the use of *upp* as a counterselectable marker.

A noteworthy advantage of our approach is the integration of all necessary functions into a single plasmid, simplifying gene editing compared to traditional approaches in closely related Lactobacillus species. The gene editing systems set up in both *L. acidophilus* and *L. gasseri* involve using the two-plasmid system, pTRK669 (helper plasmid) and pORI28 (integration plasmid), and a background strain lacking the *upp* gene (14, 15). In this system, the *upp* expression cassette is present in the pORI28 plasmid. The success of this approach would require sequential transformation of both plasmids, which makes transformation efficiency and particularly plasmid stability as the key bottlenecks. By consolidating these functionalities into one plasmid that is stable in *L. johnsonii*, our strategy markedly improves the speed and efficiency of gene editing in this bacterium.

Our demonstration of stable reestablishment of *L. johnsonii* KR09 in the murine gut following antibiotic-induced microbiota disruption underscores the utility of the engineered strain for future host-microbe interaction studies. *L. johnsonii MR1* has been extensively studied for its host immunomodulatory effects. When introduced orally into mice, *L. johnsonii* can affect mucosal immunity, systemic immunity, and intestinal function (15, 16, 30). *L. johnsonii* MR1 supplementation during the weaning period was shown to markedly alter the developing microbiome of the neonatal offspring and decrease subsequent T helper 2 (Th2) immune responses to viral infection (16). By developing this genetic system in a rodent isolate and validating the colonization ability of the engineered strain in animal models, we are now positioned to investigate the mechanisms underlying the immunomodulatory effects associated with this strain.

The establishment of this markerless gene replacement system, leveraging counterselectable markers, represents a significant advancement for functional genomics in *L. johnsonii*. While the transformation protocol and gene editing toolkit remain to be tested in other *L. johnsonii* strains, we expect this approach to work for other phylogenetically related rodent strains of *L. johnsonii*. However, we expect the host specificity of this species to influence the protocol’s generalizability across all strains. Nonetheless, the addition of this system to the *L. johnsonii* gene editing toolbox is expected to reduce the complexity and time required to assemble strain-specific resources.

Collectively, these findings establish a robust genetic platform for *L. johnsonii*, enabling precise mechanistic investigation of gene function and facilitating detailed exploration of both microbe-microbe and host-microbe interactions.

## MATERIALS AND METHODS

### Bacterial strains, plasmids, and culture media

Bacterial strains and cloning plasmids are listed in Table 1. All subcloning experiments were done in *Escherichia coli* Top10. *E. coli* strains were grown in Luria–Bertani (LB) broth or solid agar (BD Difco, 244620/244520) and incubated at 37°C with rotary shaking at 200 rpm. Twenty micrograms per milliliter of 5-bromo-4-chloro-3-indolyl β-d-galactopyranoside (X-gal) (Thermo Scientific, R0404) was added to *E. coli* LB growth media when required for blue-white colony screening. Erythromycin at a concentration of 180 µg/mL and 200 µg/mL was added to LB growth media when required for selection and maintenance of transformed strains, respectively. *L. johnsonii* strains were grown in de (MRS) broth or solid agar (BD Difco, 1813625/288210) and incubated at 37°C, anaerobically under static conditions. Sugar-free MRS media for carbon utilization studies were prepared as follows: 5 g Bacto Peptone (Gibco, 211677), 2.5 g sodium acetate (Sigma-Aldrich, S2889), 2 g Bacto Yeast Extract (Gibco, 212750), 1 g tri-ammonium citrate (Bioworld, 40100312-1), 0.1 g Magnesium sulfate (Sigma-Aldrich, 230391), 25 mg of manganese sulfate (Sigma-Aldrich, 221287), and 0.4 mL of Tween 80 (Sigma-Aldrich, P8074) in a 500 mL. Sugar-free MRS media were supplemented with 10% D-lactose (MP Biomedicals, 02199677-CF) for phenotype screening. Recombinant *L. johnsonii* strains containing plasmid or integrative mutants were cultured in MRS supplemented with either 3 µg/mL of erythromycin (Sigma-Aldrich, E0774) or 100 mg/mL of 5-Fluorouracil (Sigma-Aldrich, F6627) and incubated anaerobically at 37°C or 42°C, respectively.

**TABLE 1:** Bacterial strains and plasmids used in this study

Strain or plasmid	Genotypes or characteristics	Reference or source
*L. johnsonii* strains		
MR1	Murine isolate	18
KR09	MR1 carrying an in-frame deletion of 555bp in the *upp* gene	This study
Δ*lacLM*	KR09 carrying an inframe deletion of 2707bp in the *lacLM* genes	This study
*E. coli*		
Top10	Used as a cloning intermediate	
Plasmids		
pG+Host9	Low copy number plasmid carrying a temperature sensitive *repA*, Erm^R^	32
pMZ7	pORI28 derivative with *upp* gene inserted into the backbone	35
pBlueScript II SK(+)	Standard cloning vector, with *lacZ* for blue-white screening	
pLEM415-ldhL-mRFP1	High copy number plasmid, expressing monomeric red fluorescent protein, Erm^R^	28
pEF1-Pls3-sfGFP	Carries an IPTG-inducible GFP, Erm^R^	29
pG+DualMarker3	*upp* expression cassette and *lacZ* derivative of pG+Host9	This study

### Spot plating

Spot plating to determine sensitivity or resistance was set up as follows. A fresh culture of *L. johnsonii* was inoculated from an overnight culture and grown anaerobically, at 37°C till an OD 600 of 0.8–1. The culture was then diluted to reach an OD 600 of 0.5. This culture was then serially diluted and spot-plated on MRS plates or MRS plates supplemented with erythromycin, 5-FU, or DMSO. Plates were incubated anaerobically at 37°C overnight and then imaged.

### Plasmid construction

The pG+DualMarker3 plasmid was generated using Gibson assembly. Fragments for generating the plasmid were amplified from pG+Host9 (backbone, GibpGhost9F, and GibpGhost9R), pMZ7 (*upp* expression cassette, GibuppF, and GibuppR), and pBluescriptII SK(+) (LacZ for blue-white screening, GibLacZF2, and GibLacZR2) as shown in (Fig. 6A). pMZ7 was a gift from Paul Blainey (Addgene plasmid # 223180; http://n2t.net/addgene:223180; RRID:Addgene_223180) and pBluescriptII SK(+) was a gift from Dr. Amy Chang. Primers were purchased from Integrated DNA Technologies (IDT, Coralville, Iowa). PCR amplifications for cloning products were performed using Q5 high-fidelity PCR DNA polymerase (NEB, M0491S) in a BioRad T100 Thermocycler. DNA fragments were purified from agarose gels using Monarch DNA Gel Extraction Kit (NEB T1120S) or cleaned up using Monarch Spin PCR & DNA Cleanup Kit (NEB T1130S). The Gibson reaction was set up using NEBuilder HiFi DNA Assembly Master Mix (NEB, E2621S) as recommended by the manufacturer.

All constructs for gene deletion and insertion were generated using double digestion and ligation. The knockout/insertion cassette was created using overlap extension PCR as described here (36) and contained 400–600 bp of the upstream and downstream sequence. Genomic DNA (gDNA) of strains was purified using the DNeasy Blood & Tissue Kit (Qiagen, 69504) following the manufacturer’s instructions. Primers used for amplifying the fragments are listed in Table 2. Restriction sites for EcoRI and PstI were introduced in the 5′ end and 3′ end of the cassette, respectively, during PCR amplification. DNA restriction enzymes were purchased from New England Biolabs (NEB, R3101S, and R3140S) and were used as recommended by the manufacturer.

**TABLE 2:** PCR primers used in this study

Primer name^*a*^	Primer sequence (5′–3′)^*b*^
pG+DualMarker9	
GibLacZF2	gcgcattaacggaataaagtcagtgagcgaggaagc
GibLacZR2	gggctggcttaacaccgtctatcagggcgatg
GibpGhost9F	agttcattatcaaccaagatcctctagagtctagggacctctt
GibpGhost9R	cttcctcgctcactgactttattccgttaatgcgccatgaca
GibuppF	gccctgatagacggtgttaagccagccccgacac
GibuppR	tccctagactctagaggatcttggttgataatgaactgtgctgattacaaaaatact
*upp* deletion	
Upp_downF	acccgttgatccaacataaggcaggggatagattgttcgga
Upp_downR	GATCGAATTCtggtgatttgacaaggatatggtcat
Upp_upF	ATGCTGCAGgtgccaaaggctcctggta
Upp_upR	ccgaacaatctatcccctgccttatgttggatcaacgggtgattc
uppdelConfF	tgaatttaaggatgacttagcaggt
uppdelConfR	ctaagaggagcaaagctgca
*lacLM* deletion	
lacLMdownF	acatctgtcccccattctggatcatctagccatcga
lacLMdownR	CAGTCAGAATTCcagtgcaatcgtaggacgaat
lacLMuppF	CAGTCACTGCAGaatgccgtcagccttgt
lacLMuppR	atggctagatgatccagaatgggggacagatgttgaag
lacLMDelConf_F	atagtgtaagggatgtcaatgctagt
lacLMDelConf_R	tggtccgactccaccaat
*lacLM* insertion	
lacLMuppF	CAGTCACTGCAGaatgccgtcagccttgt
lacLMdownR	CAGTCAGAATTCcagtgcaatcgtaggacgaat

^*a*^ F, forward; R, reverse.

^*b*^ Restriction enzyme sites are underlined.

Chemically competent *E. coli* Top10 cells were prepared as described here (37) and used as the cloning intermediate. Colony PCR screening was done using Promega GoTaq Master Mix (Promega, M7122). Plasmid DNA was purified using the E.Z.N.A. Plasmid DNA Mini Kit (Omega Bio-tek Inc, D6942-01) or Plasmid Midi Kit (Qiagen, 12143). All plasmids generated were verified by whole plasmid sequencing by Plasmidsaurus using Oxford Nanopore Technology with custom analysis and annotation.

### Transformation in *L. johnsonii*

Electrocompetent *L. johnsonii* cells were prepared as follows: 3× SMEB buffer was used as the electroporation buffer: 30.6 g sucrose (Sigma-Aldrich, S9378) and 3 mL of 100 mM MgCl2 (Sigma-Aldrich, M8266) in 100 mL distilled water, followed by filter sterilization; 10 mL of MRS was inoculated with *L. johnsonii* from glycerol stock and incubated overnight anaerobically at 37°C under static growth conditions. Three milliliters of the overnight culture was then used to inoculate 100 mL of pre-warmed 2% glycine MRS media. The culture was incubated aerobically at 30°C under static growth conditions until it reached an OD 600 of 0.4–0.5. The culture was then incubated in ice for 5 min and spun down at 3,000 rpm for 10 min at 4°C. The cells were then washed twice with ice-cold 3× SMEB buffer and spun down at 2,000 rpm for 10 min at 4°C. The final pellet was resuspended in 4 mL of ice-cold 3× SMEB; 200 µL of competent cells were aliquoted and kept on ice for transformation or snap frozen and stored at −80°C. Electroporation in *L. johnsonii* was done as follows: 200 µL of *L. johnsonii* electrocompetent cells were mixed gently with 2–5 µg of plasmid and incubated on ice for 5 min. The mixture was then transferred to a 0.2 cm cuvette, and the cells were electroporated at 2 kV, 400 ohms, and 25 µF. Successful transformation has a time constant of 5−6s. The cells were then transferred into a 1.5 mL tube containing 800 μL of pre-warmed MRS media and incubated anaerobically for 3 h. Transformants were selected by plating 100 μL of recovered cells on an antibiotic selection plate. The remaining 900 μL were concentrated into 100 μL and plated on a second plate and incubated anaerobically at 37°C for 24–48 h.

### Deletion and complementation of mutants

Deletion/insertion mutants were selected as described in Fig. 3C. Following the transformation of deletion/insertion plasmid constructs in *L. johnsonii,* colonies were screened using colony PCR to confirm transformation. Next, positive colonies were grown anaerobically at 37°C in MRS, supplemented with 3 µg/mL erythromycin to maintain the plasmid in the cells. The first crossover event was triggered by sub-culturing these cells (1:100) twice, anaerobically at a high temperature of 42°C in MRS, supplemented with 3 µg/mL erythromycin. Following 48 h of growth under these conditions, the second crossover was encouraged by sub-culturing the cells at 42°C in MRS without the antibiotic stress. The culture was left to grow under these conditions for 3–4 h, after which it was serially diluted and plated on MRS plates supplemented with 5-FU. Colonies that grow on the counterselectable plate no longer have the plasmid. They were then screened for deletion/insertion using colony PCR.

### BIOLOG assay

PM1 (Biolog, 12111) and PM2A (Biolog, 12112) phenotype microarrays from BIOLOG were used to test the carbon utilization of *L. johnsonii* strains. *L. johnsonii* strains from glycerol stocks were inoculated in MRS media and incubated overnight, anaerobically at 37°C. Reduced sugar-free MRS media were inoculated with 1% of the overnight culture. The freshly inoculated culture was then aliquoted into the PM1 and PM2A plates, with 140 µL per reaction well. The plates were all set up anaerobically and sealed before being moved into ODIN. The plates were incubated at 37°C, and OD 590 readings were collected every 20 min for 24 h.

### Antibiotic treatment and *L. johnsonii* KR09 gastric colonization

C57BL/6J mice (Jackson Laboratories, Indianapolis, IN) were housed 5 mice to a cage under specific-pathogen-free conditions in enclosed filter-top cages. The experiment was performed using two cages per treatment condition and five mice per cage. All mice were housed in the same room. Food and sterile water were given *ad libitum*. Food remained constant throughout the experiment to minimize the effect of diet on the microbiota. The experiment was set up as described in Fig. 8A. Ceftriaxone (0.5 mg/mL) (Sigma, C5793) was administered orally to mice *ad libitum* in drinking water for 4 days as previously described (31). Antibiotic water was replaced every other day. *L. johnsonii* KR09 was grown in MRS media, anaerobically overnight at 37°C. For gavage, the culture was washed once with PBS and resuspended to a final concentration of 5 × 10^8^ CFU/mL in PBS. Mice were inoculated with the bacterium by oral administration using a 24-gauge feeding needle attached to a 1-mL syringe. The syringe containing *L. johnsonii* KR09 was mounted on a Stepper repetitive pipette (Tridak, Brookfield, CT) to deliver an equivalent amount of inoculum, 200 µL, to each mouse. Fecal samples were collected at regular intervals, as described in the schematic. Collected samples were homogenized in 1 mL of PBS, followed by serial dilution and plating. Samples were plated on MRS agar or MRS agar supplemented with 100 µg/mL of 5-FU to evaluate CFU levels of total *L. johnsonii* and *L. johnsonii* KR09. Following euthanasia, tissue samples from the stomach, small intestine (duodenum, jejunum, and ileum), cecum, and colon were collected for CFU plating and microbiome analysis.

### 16S rRNA sequencing and microbiome analysis

Procedures were performed as previously described in detail (38). Genomic DNA was extracted using DNeasy Blood and Tissue Kit (Qiagen, 69504) and homogenized in bead beating tubes (Revvity, 19-624). Raw cecal slurries were processed in duplicate. The V4 region of the 16S rRNA gene was amplified according to protocol, and sequencing was performed on the Illumina MiSeq platform (San Diego, CA) as previously described (38). AccuPrime High-Fidelity Taq was used, with PCR cycling conditions as follows: 95°C for 2 min, then 20 cycles of touchdown PCR followed by 20 cycles of standard PCR, and finally at 72°C for 10 min. Sterile water, DNA isolation controls, and empty wells were used as negative controls. Synthetic standard bacterial communities (Zymo Research, Irvine, CA; catalog no. D6306) were used as positive controls. Sequence data were processed and analyzed using the software Mothur v.1.43.0 according to the Standard Operating Procedure for MiSeq sequence data using a minimum sequence length of 250 base pairs. A shared community file and a phylotyped (genus-level grouping) file were generated using operational taxonomic units (OTUs) binned at 97% identity in Mothur. OTU numbers were arbitrarily assigned in the binning process and are referred to throughout the manuscript in association with their most specified level of taxonomy. Classification of OTUs was carried out using the Mothur implementation of the Ribosomal Database Project (RDP) Classifier and the RDP taxonomy training set 14 (Trainset14_032015.rdp), available on the Mothur website. Data analyses and figures were generated in R (v4.3.2).

## Data Availability

Sequences used in the 16S rRNA analyses are available via the NCBI Sequence Read Archive under accession number PRJNA1459492.

## SUPPLEMENTAL MATERIAL

Supplemental figures jb.00100-26-s0001.docx Figures S1 to S3.

ASM does not own the copyrights to Supplemental Material that may be linked to, or accessed through, an article. The authors have granted ASM a non-exclusive, world-wide license to publish the Supplemental Material files. Please contact the corresponding author directly for reuse.
