# Supplementary material for: Development of a gene-editing strategy to overcome genetic intractability in Lactobacillus johnsonii
Source: J Bacteriol. 2026 Jun 29;208(7):e00100-26. doi: 10.1128/jb.00100-26 (PMC13393412; doi:10.1128/jb.00100-26)
Supplement: Supplemental figures — Figures S1 to S3. [file jb.00100-26-s0001.docx]

**Supplemental material**

**
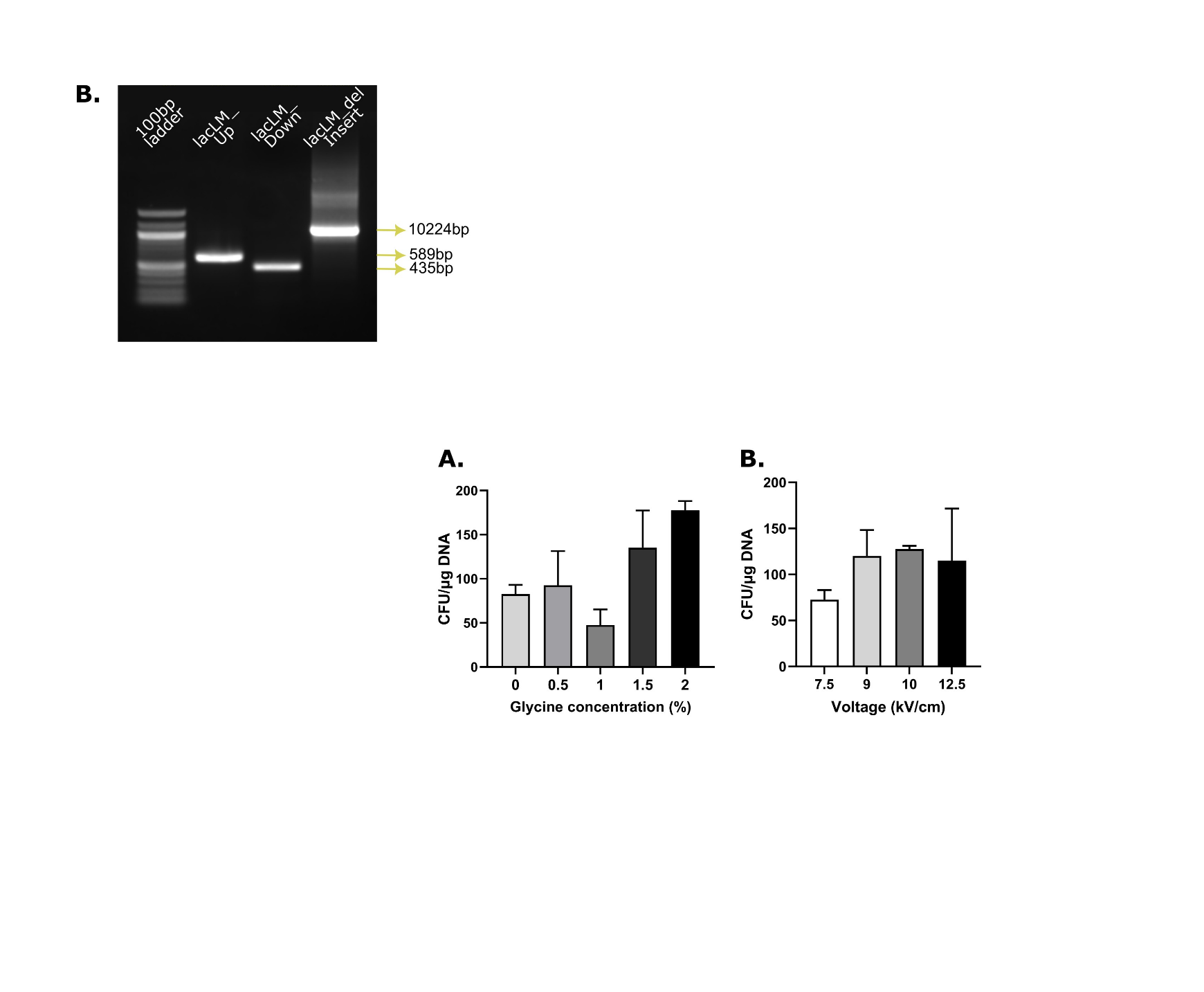
**

**Supplemental Figure 1 | Optimization of transformation procedure for *L. johnsonii.*** Different glycine concentrations ranging from 0 – 2% were tested in the growth media (A). Similarly, different electroporation voltage settings were also tested (B). 2% Glycine concentration and 2 kV in a 0.2 mm cuvette had the highest transformation efficiency. Transformation efficiency was tested using the pG+Host9 plasmid. Experiments were done in duplicates.


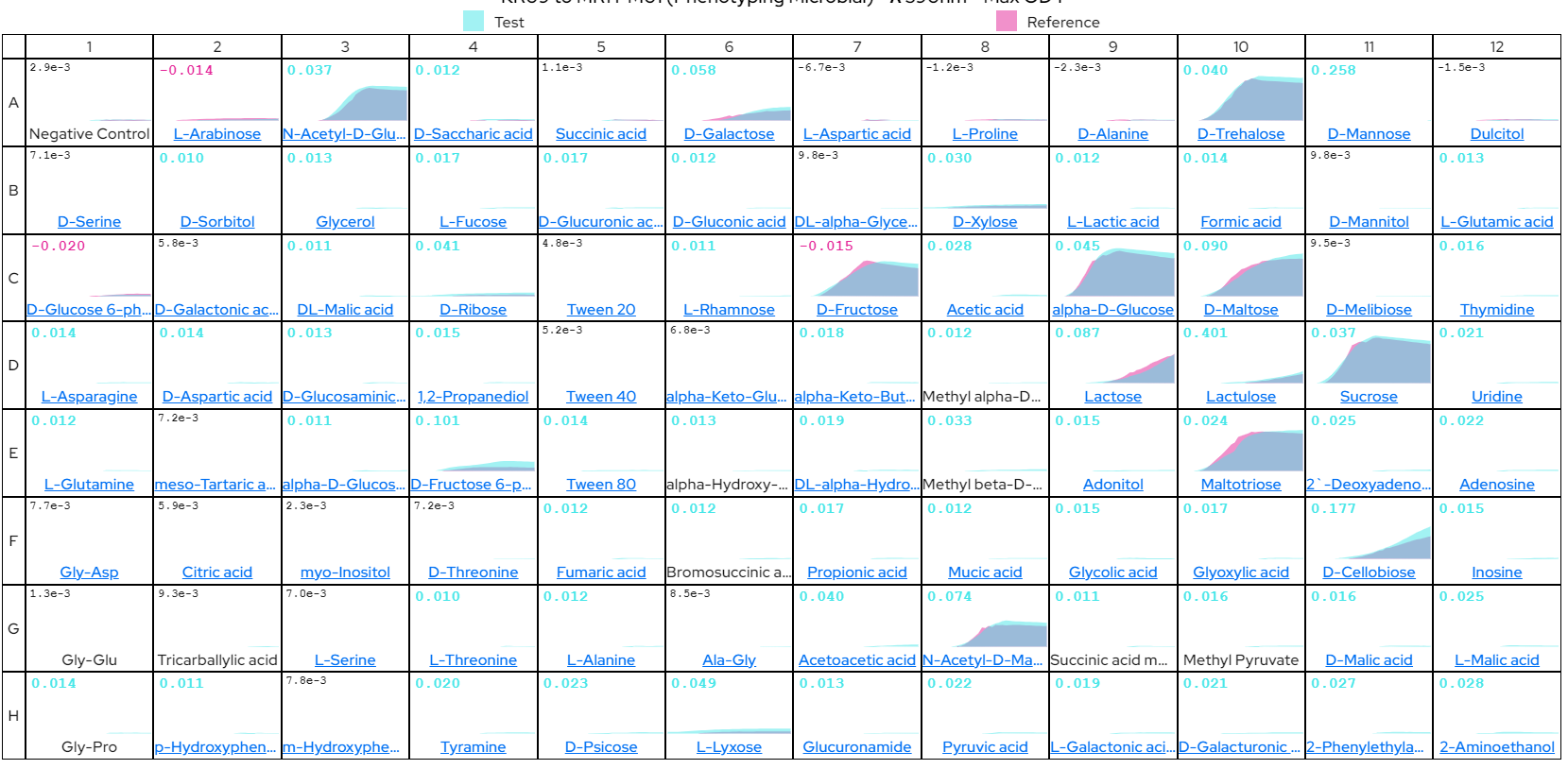


**Supplemental Figure 2 | Biolog carbohydrate utilization plate PM1 analysis** for *L. johnsonii* MR1 (Reference) and *L. johnsonii* KR09 (Test). OD 590 readings were collected over a 12 hr time period.

**
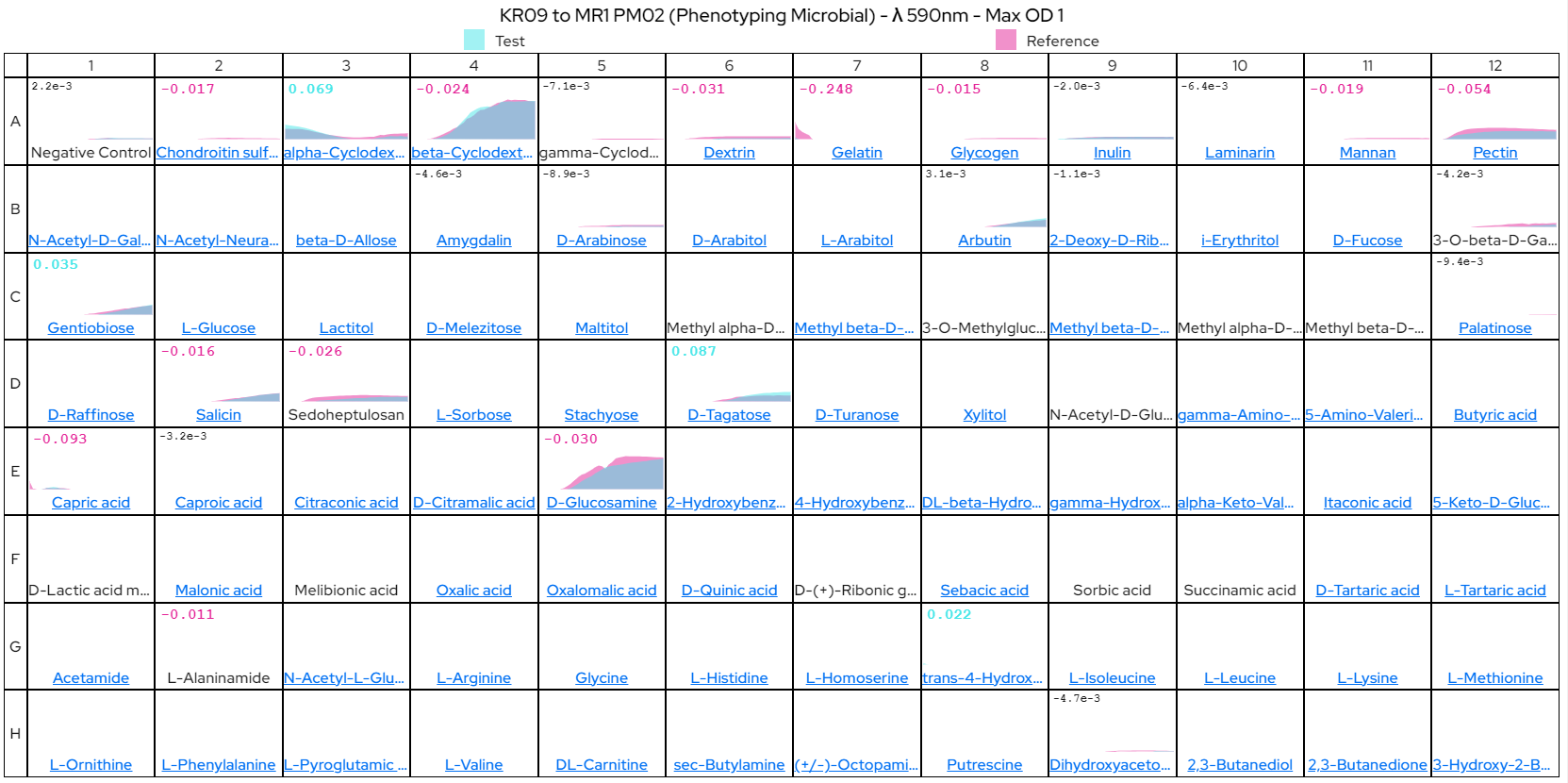
**

**Supplemental Figure 3 | Biolog carbohydrate utilization plate PM2 analysis** for *L. johnsonii* MR1 (Reference) and *L. johnsonii* KR09 (Test). OD 590 readings were collected over a 12 hr time period.
